# *In vitro* combination therapy of pathologic angiogenesis using anti-vascular endothelial growth factor and anti-neuropilin-1 nanobodies

**DOI:** 10.22038/ijbms.2020.47782.11000

**Published:** 2020-10

**Authors:** Nastaran Mohseni, Reyhaneh Roshan, Shamsi Naderi, Mahdi Behdani, Fatemeh Kazemi-Lomedasht

**Affiliations:** 1 Venom and Biotherapeutics Molecules Laboratory, Biotechnology Department, Biotechnology Research Center, Pasteur Institute of Iran, Tehran, Iran

**Keywords:** Cancer, Dual targeting, Nanobody, NRP-1, Single domain antibody, VEGF

## Abstract

**Objective(s):**

Emergence of resistant tumor cells to the current therapeutics is the main hindrance in cancer treatment. Combination therapy, which mixes two or more drugs, is a way to overcome resistant problems of cancer cells to current treatments. Nanobodies are promising tools in cancer therapy due to their high affinity as well as high penetration to tumor sites.

**Materials and Methods:**

Here, the inhibitory effect of mixtures of two nanobodies (anti-vascular endothelial growth factor (VEGF) and anti-neuropilin-1 (NRP-1) nanobodies) on tube formation of human endothelial cells *in vitro* and *ex vivo* were analyzed.

**Results:**

Results showed that combination of two drugs significantly inhibited proliferation and tube formation of human endothelial cells. In addition, mixtures of two nanobodies inhibited angiogenesis in chick chorioallantoic membrane (CAM) assay efficiently compared with each individual nanobody.

**Conclusion:**

Results highlight the efficacy of combination therapy of cancer compared with mono-therapy and promises development of novel anti-cancer therapeutics based on nanobodies targeting two or more targets of tumor cells.

## Introduction

Angiogenesis has an important role in the development of a malignant tumor. Angiogenesis is the physiological process characterized by the formation of new blood vessels. Angiogenesis plays an important role in the migration, growth, and differentiation of endothelial cells. In addition, it plays a key role in the development of diseases such as cancer and inflammation. In the signaling pathway, vascular endothelial growth factor (VEGF) is crucial for the growth of the vessels. VEGF, indicates a family of glycoproteins that includes seven members (VEGF-A, VEGF-B, VEGF-C, VEGF-D, VEGF-E, VEGF-F, and placental growth factor (PlGF)) (1). In the signaling pathway, molecules playing critical roles in angiogenesis are angiopoietin (Ang)–Tie, and Ephrin/Eph receptor Notch. VEGF and its receptors (VEGFRs) have been shown to play an important role in the development of tumors and metastasis (1). It should be noted, VEGFR-1 plays the main role in physiological and pathological angiogenesis, including tumor angiogenesis (2). VEGFR-2 is a receptor with high affinity for VEGF, and the most important angiogenic growth factor receptor in angiogenesis. VEGFR-2 (also known as KDR/fetal liver kinase, Flk-1 eor kinase-insert domain receptor) able to bind VEGF-A, C, and D. VEGFR2 is the major signal transducer of VEGF in endothelial cells (3, 4). The VEGFR-2 signaling pathway plays the main role in VEGF effects during vasodilatation, proliferation, as well as migration of endothelial cells (5).  In addition, Neuropilin-1 (NRP-1) is a single-pass transmembrane protein that acts as a cell surface receptor for VEGF165 (one of the main isoforms of VEGF) to increase endothelial cell migration during angiogenesis with VEGFR-2. Moreover, it plays an important role in VEGF-induced vascular permeability and arteriogenesis. Additionally, NR-1 activates intracellular kinase ABL1 in VEGF-independent signaling. Semaphorin-Neuropilin-1 signaling affects vessel maturation and vascular permeability in ocular disease (6).  As mentioned above, VEGF and its receptors (VEGFR-1, VEGFR-2, and Neuropilin (NRP-1)) have important roles in angiogenesis and can serve as appropriate targets in the treatment of cancer and vascular disease (7). Synergistic effects of anti-VEGF therapy with other therapeutics like NRP-1 have been evaluated in many pieces of research (8-11). Here, for the first time, the synergistic effect of an anti-VEGF nanobody with anti-NRP-1 nanobody in inhibition of angiogenesis *in vitro* and *ex vivo* has been evaluated. 

## Materials and Methods

***Cell culture***

Human umbilical vein endothelial cells (HUVEC) were obtained from the Cell Bank of Pasteur Institute of Iran. HUVECs were maintained in HAMS-F12 medium supplemented with 10% fetal bovine serum (FBS) (Gibco) and antibiotics (100 IU/ml penicillin G, 100 μg/ml streptomycin), (Gibco). HUVECs was maintained at 37 °C in an environment of 5% CO_2_. Geltrex^TM ^LDEV-Free Reduced Growth Factor Basement Membrane M was purchased from Gibco. 

***Preparing of nanobodies***

Nanobodies against VEGF (12. 13) and NRP-1 (unpublished) were obtained from our previous studies. A possible additive effect of anti-angiogenesis was tested with combination of two nanobodies. The nanobodies were expressed and purified as described in a previous work (12). Due to similar affinity of both nanobodies (in the nanomolar range), different concentrations (2.5, 5, 10, 20 µg/ml) of each nanobody were prepared separately. Next, equal concentrations of both nanobodies (anti-VEGF nanobody and anti-NRP-1 nanobody) were mixed and used for *in vitro* and *ex vivo* assays. 

***In vitro assays***

*Proliferation inhibition assay*

Anti-angiogenesis effects of the mixture of two nanobodies were investigated using MTT assay. The MTT assay was performed using 3-(4, 5-dimethylthiazol-2-yl)-2, 5-diphenyltetrazolium bromide. HUVEC cells were seeded (3× 10^3^ cells/well) into a 96-well plate. After cell growth, treatment was performed on HUVEC cells. So that, two nanobodies at different concentrations (2.5, 5, 10 µg/ml) were mixed and PBS as control was added to each well for 24 and 48 hr. Then, 10 μl MTT solution (5 mg/ml) was added to every single well and incubated at 37 °C. After 4 hr, the medium was removed and 100 μl of dimethyl sulfoxide (DMSO) was added to each well to dissolve the resulting MTT-formazan crystals. The absorbance of formazan was measured at 570 nm using a plate reading spectrophotometer. 

*Tube formation assay*

To determine *in vitro* capability of capillary formation of anti-VEGF or anti-NRP-1 nanobody, tube formation assay was performed. Matrigel Basement Membrane Matrix (50 μl) was coated in 96-well plates at 37 ^°^C for 1 hr. Then, 4 × 10^3^ HUVEC cells with 10 µg/ml of anti-VEGF or anti-NRP-1 nanobody or a mixture of two nanobodies or PBS (as negative control) were added on Matrigel. The tube formation assay was evaluated after 1 to 6 hr by an inverted microscope. Tube-like structures were analyzed and quantified by Image J software.

***Ex vivo assay***

*CAM assay*

Fertilized pathogen-free eggs were carefully sterilized with isopropanol 70% and incubated at 37 ^°^C and 60 % relative humidity with gentle movement. On day 6, eggs were removed from the incubator and then the sides of the eggs, where the embryos had been located, were marked by a pencil. The opposite of the mark was broken and the contents of the egg were transferred into a petri dish so that the embryo is specifically placed on the egg yolk. 

Then autoclaved discs containing concentrations of a mixture of two nanobodies (10 μg/ml of anti-VEGF nanobody + anti-NRP-1 nanobody) or each single nanobody (10 μg/ml of anti-VEGF nanobody or anti-NRP-1 nanobody) or 10 µl PBS (as negative control) were placed on top of the embryo and the petri dish was transferred to the incubator for 24 hr. Finally, inhibition of angiogenesis was observed macroscopically. 

***Statistical analysis***

Statistical analyses were performed using the GraphPad Prism software package (GraphPad, San Diego, CA). A *P*-value of 0.05% or lower was considered statistically significant.

## Results

***Cytotoxicity***

**Figure 1:**
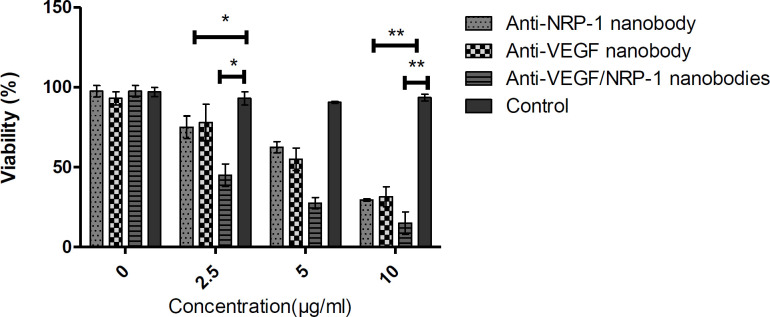
Cytotoxicity of combination of two nanobodies on human endothelial cell proliferation. Higher concentrations of two drugs result in a lower percentage of viable cells. * :* P*-value<0.05, **: *P*-value<0.001 . The experiment was performed in triplicate and represented as mean±SD

The cytotoxic effects of the mixture of two nanobodies as well as each single nanobody on HUVEC cell viability were determined by MTT assay. Our results demonstrated a reduction in cell viability in a time and dose-dependent manner after 24 and 48 hr (Figure 1). Results demonstrated that at the lowest concentration of the mixture of anti-VEGF/NRP-1 nanobody (2.5 μg/ml), about less than half of cells survived (45.5%). Our results determined that the mixture of two nanobodies affected HUVEC cell viability even at low concentrations (2.5 μg/ml).

***Tube formation assay***

**Figure 2:**
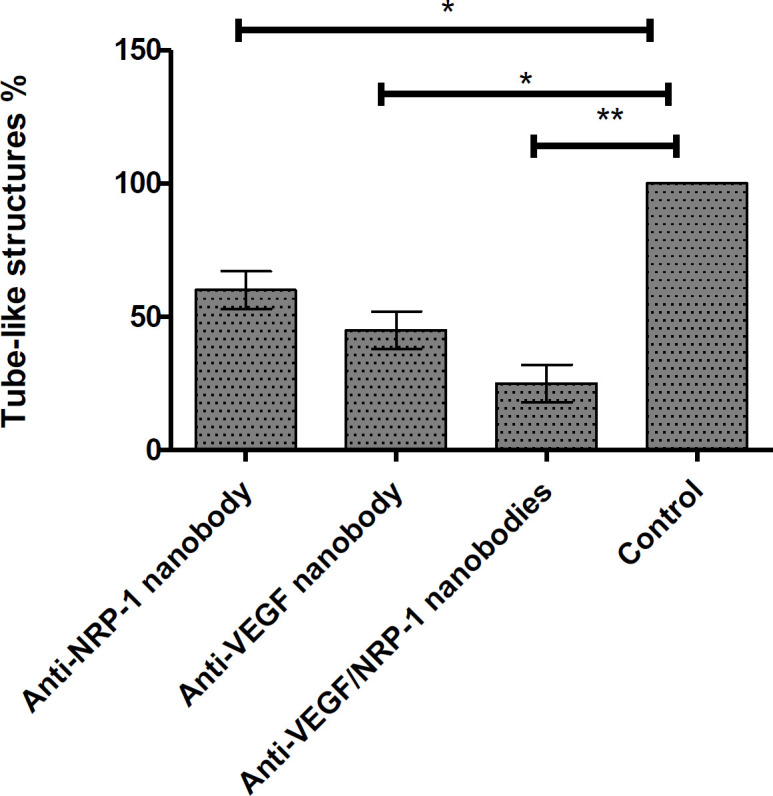
Tube formation results. As shown, percent of tube-like structures were 60, 45, and 25% in case of anti-NRP-1 nanobody, anti-VEGF nanobody, and mixtures of both nanobodies, respectively. Control well-formed complete tube-like structures on Matrigel assay. * : *P*-value<0.05, **: *P*-value<0.001. The assay was performed in triplicate and data presents mean± SD

To determine the capability of capillary formation of the mixture of two nanobodies as well as single nanobodies *in vitro*, tube formation assay was performed. Results showed that mixtures of two nanobodies as well as each single nanobody significantly inhibited tube formation of HUVEC cells on. However, the effect of mixture drugs was more than each single nanobody on tube formation of HUVEC cells (Figure 2). 

***CAM assay***

**Figure 3:**
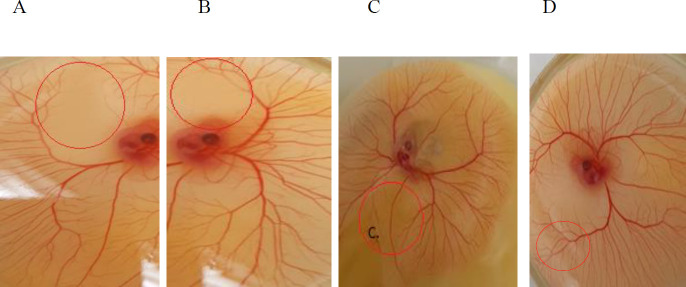
Chick chorioallantoic membrane assay results. A: anti-NRP-1 nanobody, B: anti-VEGF nanobody, C: mixtures of both anti-VEFG/NRP-1 nanobodies, D: control. Anti-VEFG/NRP-1 nanobodies inhibited angiogenesis *ex vivo* higher than each single nanobody. Chick embryo images represent treatment with two drugs after 48 hr

 Anti-angiogenic activities of two drugs were confirmed by using the CAM assay. As illustrated in Figure 3treatment with two drugs for 24 and 48 hr showed a dose-dependent reduction in the number and length of the branching of new vessels compared with the control group. Based on the results, at a concentration of 10 μg/ml of anti-VEGF/NRP-1 nanobody vessels formation was inhibited significantly. 

## Discussion

Considering the importance of cancer and attempting to control it, today, several studies have been tried to control and treat cancer. Most studies have focused on eliminating tumor tissue, in which inhibiting tumor growth, invasion, and angiogenesis are of particular importance. In adulthood, angiogenesis has an important role in the physiological process. Angiogenesis requires sufficient action between multiple growth factors and cell-adhesion molecules (14). The present studies show that NRP-1 plays a key role in angiogenesis in association with VEGF and its receptor. It also plays a role in tumorigenesis by forming NRP-1 / VEGF / VEGFR2 complex (15). NRP-1 is often expressed in carcinoma (in particular tumors of epithelial origin) including lung, chest, prostate, pancreas, and clone carcinoma. Excessive expression of NRP-1 is associated with progression of disease as well as tumor metastasis (16). Studies showed that inhibition of the NRP-1 pathway results in prevention of the new blood vessels formation (17-19). The effect of NRP-1 on cancer progression is often attributed to increased activation of VEGFR2 in response to VEGF. Although, in some tumors, NRP-1 is expressed without the expression of VEGFR1 and VEGFR2. In such a situation, the role of VEGFRs becomes dimmed (20). However, the formation of a triple complex of NRP-1, VEGFR2, and VEGF165 has been proven to promote VEGF signaling in numerous experiments. Inhibition of VEGF binding to NRP-1 receptor in endothelial cells leads to a decrease in VEGF binding to VEGFR2, resulting in decreased cell proliferation. Accordingly, in this study, we investigated the *in vitro* and *ex vivo* characteristics of specific nanobodies towards VEGF/NRP-1 in a dual targeting. We demonstrated that the mixture of two nanobodies had an additive effect in the inhibition of angiogenesis. It has been shown that the simultaneous transfection of VEGFR2 and NRP-1 into endothelial cells increases VEGF binding to VEGFR2 and increases cell proliferation compared with when cells express VEGFR2 alone. Endothelial cells expressing NRP-1 alone and not expressing VEGFR2 are not capable of responding to any of the VEGF isoforms (21-23). We reported that the viability of HUVEC cells reduced in the assessment of the MTT assay. Furthermore, we showed the ability of combination therapy in inhibition of angiogenesis both *in vitro* and *ex vivo*. We previously showed the inhibitory effect of anti-NRP-1 nanobody *in vitro* and *ex vivo*. The binding specificity of anti-NRP-1 nanobody was confirmed by the angiogenesis studies on the chicken embryo. Some evidence suggests that NRP-1 may present VEGF165 to VEGFR-2 to be involved in tumor angiogenesis in endothelial cells as a positive regulator of the VEGF signaling pathway (24). Studies have shown that VEGFR-2 is a predominant receptor in VEGF-induced endothelial cell mitogenesis and angiogenic signaling (25). We demonstrated that a combination of two nanobodies (anti-NRP-1 and anti-VEGF nanobodies) had a profound response rate *in vitro* and *ex vivo*. Multiple studies have indicated inhibition of tumor angiogenesis might arrest tumor progression but would not eradicate the tumor as a stand-alone therapy, especially with a single anti-angiogenic agent (26). Moreover, one of the limiting factors in single-agent treatment with anti-angiogenic drugs can be adaptive resistance (27). One approach that seems to be more promising in cancer research, is the use of one or more drugs in combination therapies that target more anti-angiogenic pathways. Inhibition of angiogenesis is a potentially promising pathway for cancer treatment (28). In recent years, research has been towards the field of treatment and the first drug targeting the VEGF-A pathway (anti-angiogenic agents) have been approved for clinical use (29). However, treatment with anti-angiogenic agents did not improve overall survival significantly in a wide variety of cancers (30). Many potential factors could lead to a combination of treatment effectiveness. However, the investigation is not complete. Although Bevacizumab can be described as a drug that has shown promising anti-angiogenic activity, it cannot prevent the interaction between VEGF/ RTKs and NRPs, which are key factors in the survival of cancer stem cells (31). Many studies have shown the ability of VEGF/NRP cellular signaling that affects tumor cell survival using activating the PI3K–AKT pathway (32). Several studies have investigated the combination of agents that target two angiogenic factors. According to available information, the combination regimen that significantly targets the interaction of VEGF and NRP-1 has shown promising results following anti-VEGF activity.

## Conclusion

We examined specific nanobodies towards VEGF/ NRP-1 in a dual targeting *in vitro* and *ex vivo*. We demonstrated in combination therapy, targeting of VEGF and NRP-1 simultaneously, displaying an additive response *in vitro*. The *ex vivo *CAM assay, treatment with two nanobodies (anti-VEGF/ NRP-1 nanobodies) resulted in effective decrease of angiogenesis and vessel growth. Results highlight the efficacy of combination therapy of cancer compared with mono-therapy and promise development of novel anti-cancer therapeutics based on nanobodies targeting two or more targets of tumor cells.
